# Collective Impact on Prevention: Let's Talk About Children Service Model and Decrease in Referrals to Child Protection Services

**DOI:** 10.3389/fpsyt.2019.00064

**Published:** 2019-02-18

**Authors:** Mika Niemelä, Hannu Kallunki, Jaana Jokinen, Sami Räsänen, Birkitta Ala-Aho, Helinä Hakko, Tiina Ristikari, Tytti Solantaus

**Affiliations:** ^1^Center for Life Course Health, Research, University of Oulu, Oulu, Finland; ^2^Department of Psychiatry, Oulu University Hospital, Oulu, Finland; ^3^Research Unit of Clinical Neurosciences, Department of Psychiatry, University of Oulu, Oulu, Finland; ^4^Joint Municipal Authority of Wellbeing in Raahe District, Raahe, Finland; ^5^Faculty of Social Sciences, University of Lapland, Rovaniemi, Finland; ^6^Adolescents and Families Unit, National Institute for Health and Welfare Children, Oulu, Finland; ^7^Finnish Association for Mental Health, Helsinki, Finland; ^8^Mental Health Unit, National Institute for Health and Welfare, Helsinki, Finland

**Keywords:** collective impact, child protection services, family adversity, Let's Talk about Children intervention, Let's Talk about Children Service Model, parental mental health disorders, prevention

## Abstract

**Background:** Families with parental mental health issues often have numerous problems needing multilevel measures to address them. The “Let's Talk about Children Service Model (LT-SM)” is a community-based service approach aiming at collectively impacting population needs regarding child protection services. Three municipalities in the Raahe District (RD) of Finland requested implementation of the LT Service Model. This paper describes the model and first results.

**Methods:** The LT Service Model connects relevant stakeholders with families and their social networks aiming at the shared goal of supporting children's everyday life at home, kindergarten, school, and leisure environments. Parents, teachers, and other caretakers are supported by LT interventions. An infrastructure for collaboration, decision making, monitoring, training, and feedback is established, embracing health, social and educational services, and other stakeholders. Referrals to child protection services were compared with national data before (2009–2013) and after implementation of the LT Service Model (2013–2016). Analyses were conducted using the joinpoint regression method.

**Results:** There was a significant decrease in the underage population referred to child protection services in RD (AAPC = −6.9; *p* = 0.013) between 2013 and 2016, in contrast with an increased rate nationwide (AAPC = 1.9; *p* = 0.020).

**Conclusion:** In the LT Service Model, prevention starts in children's everyday life as the uniting, common goal for multiple stakeholders and an integrated service structure is developed to support this effort. The first results are promising, showing an appreciable decrease in referrals to child protection services, although further research with longer follow-up and across other municipalities is needed.

## Introduction

Intergenerational transfer of parental problems and family adversity is an individual, family, and societal concern. In Finland, parental mental health and substance abuse problems, often associated with other adversities, are also major reasons for child protection measures (1). Family adversity, as well as family resilience, is multi-determined and embedded in the social ecology of the family and society (2). Multilevel prevention is necessary (3) and comprehensive approaches are needed to achieve a collective impact on population-level outcomes (4).

The “Let's Talk about Children Service Model (LT-SM)” is a community-based model for promoting child and family well-being and resilience and preventing family and child dysfunction. It is aimed to overcome the fragmentation of services and lack of common goals, which have been major obstacles for integrated approaches in health, social, and educational services (4). The core of LT-SM is supporting children in their everyday life at home, kindergarten, school, and leisure environments (children's developmental contexts). Parental and family problems interfere with everyday routines and interactions with a risk of compromising the child's well-being and development (3). In LT-SM, health and social services, as well as kindergartens, schools, and available non-governmental organizations, commit to a shared goal: to support, in collaboration with others, children's everyday life in all developmental contexts and to build the corresponding service structure.

Within this model, LT interventions (5–8)^1^ are used with families; LT is a relational, cognitive, and behavioral intervention approach aiming to support parents, teachers, and other caretakers in their everyday interactions and activities with children. It is used within mental health and substance use services as well as in cases of severe somatic conditions. In kindergartens and schools, LT for Schools and Early Education (mielenterveysseura.fi) is used universally. The focus is to enhance sensitive and supportive interactions with the child as well as mutually supportive parent-teacher relationships based on a shared understanding of the child at home and school. Children participate in this program depending on their age.

LT Intervention 2018 (www.mielenterveysseura.fi) includes two steps. LT-Discussion depicts the child's ordinary day in all developmental contexts and an action plan is made with parents to enhance the identified strengths and to give support in vulnerabilities; this step is performed in one or two semi-structured meetings. When needed, the second step LT-Network meeting is planned with parents and convened to carry out the necessary support with the family's social network, services, and possible others. LT-Network meeting provides a forum for case-based cross-sectoral collaboration, including also the families and their social network.

RD decided to implement LT-SM in 2011 with the purpose to increase family and child well-being indicated by a reduction in referrals to child protection services (9, 10). It was hypothesized that there would be a decrease in the referrals after an initial increase due to revealing latent needs and that this process would take several years to yield results (11).

The present research paper describes LT-SM and findings related to referrals to child protection services. A detailed description of the implementation of this model is beyond the scope of this paper.

RD includes an industrial town and two smaller rural communities consisting of a population of 35,000 inhabitants with 8,300 under-aged children. The unemployment rate was 10% in 2012. Finland has national health and social services that also cover RD.

## Materials and Methods

### Let's Talk About Children Service Model and Its Implementation

#### Strategy Level

Both administrative and political leaders in the health, social services, and educational sectors committed to implementing LT-SM at the turn of 2011-2012. LT-SM was incorporated into the Plan for Children's Well-being.

#### Organizational Level

##### Multiagent management group (MMG)

MMG is needed to run the implementation and develop cross-sectoral collaboration. This group includes leaders from all relevant sectors. One of its main functions is to report the progress and challenges concerning the implementation of LT-SM. Issues that need to be solved are related to subjects such as decision-making, budgeting and allocating resources across sectoral divides, ethical and practical guidelines relating to reporting and monitoring, documenting patient/client/student records, and networking with families.

MMG was founded in RD in early 2013. MMG meets four times a year and also based on ongoing needs.

##### One contact service (OCS)

OCS coordinates case-based collaboration. It arranges LT-Network meetings with the necessary participants. Therefore, it also encounters challenges in collaboration and reports them to MMG. Functional feedback system allows the organization to learn from experience.

OCS was created in RD in August 2013 and is being run by two social workers. One phone call or email is enough to activate the Service. As the work of this service concerns prevention and resilience, even a timid and isolated child needing a hobby in a poor family is a valid reason for action.

#### Workforce Capacity

Different levels of the workforce, from administrative leaders to practitioners working with service users, are educated concerning the main principles, and the ethos and praxis of LT interventions and LT-SM. Method training in LT interventions (in a span of 3 days) is organized for those practitioners working with family members. Training trainers (5 days) is organized to ensure the sustainability of the model. A long-term plan for training is prepared to overcome disruptions due to changes in staff.

Method training in LT interventions started in RD in August 2012. In the first round, 30 practitioners coming from health, social services, and educational sectors were trained. Twenty-five of them also participated in the trainer's training. Dr. Niemelä was responsible for this training program.

Between 2012 and 2013, in RD method training included all 220 workers in health and social services and 270 kindergarten teachers and assistants. Within the schools, practically all employees were trained in this industrial town, including 350 teachers, assistants, and student welfare professionals, while in the two rural communities 30 special education teachers and student welfare professionals were trained.

#### Population-Level Communication

The population was informed about LT-SM and LT interventions through local media and during evening meetings with parents at the schools and kindergartens. Service users received face-to-face information and leaflets.

#### Implementation of the Model by 2013

By the end of 2013, LT-SM infrastructure was functional and LT intervention was offered to parents at the mental health and substance use services; attention was also provided to parents with severe somatic conditions. In kindergartens, LT interventions with parents were carried out yearly, and biannually in schools.

### Data and Statistical Analyses

Data regarding referrals to child welfare services from 2009 to 2016 were obtained from Sotkanet, an Indicator Bank produced by the National Institute for Health and Welfare (THL) (12). The size of the population between zero and 17 years in RD and the whole country was obtained from the PX-Web population database provided by Statistics Finland. No ethical approval was required for using combined data from open-access data sources.

With regards to time trend analyses, a joinpoint regression program and the average annual percentage change (AAPC) method (13, 14) were employed to estimate the percentage rates of the 0- to 17-year-old population referred to child protection services. The AAPC method used the underlying joinpoint regression model to calculate a summary measure above the fixed pre-specified interval. In the current study, the year 2013 was used as a cut-off year regarding the change in trends. Due to the relatively small number of referrals causing a high variability in the rates of the 0 to 17-year-old population referred to child protection services in RD compared to those of the whole country, a 3-year moving average (3-year moving average at the end of time periods) was used for the statistical modeling.

## Results

**Figure 1:**
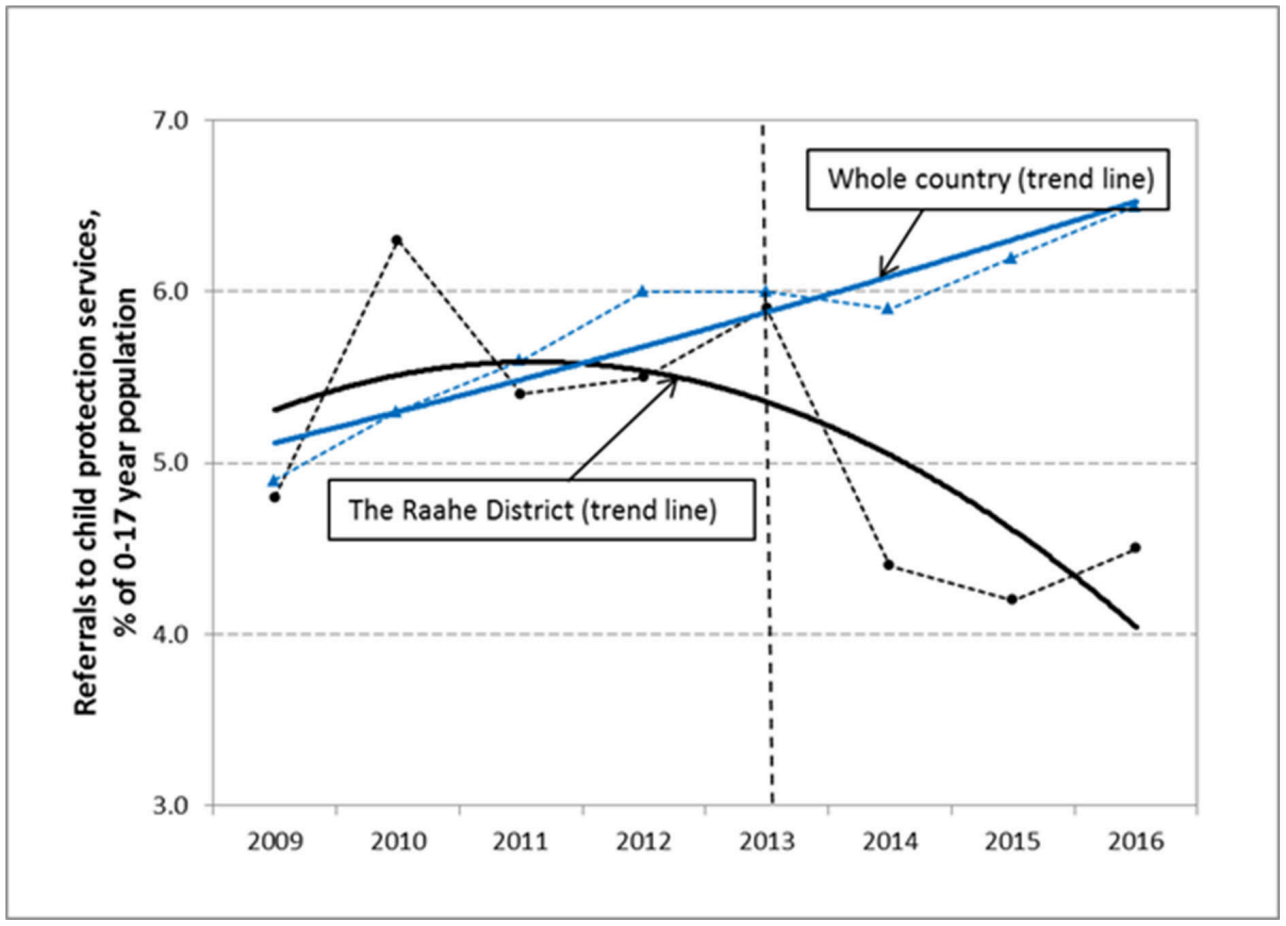
The time trends (solid lines) and the observed percentage rates (dotted lines) of referrals to child protection services among the 0 to 17-year-old population in Raahe District and the whole country. The vertical dotted line indicates the year 2013 from which the Let's Talk about Children Service Model was in the routine use in the services of RD.

Figure 1 shows the trend in percentage rates of the 0 to 17-year-old population referred to child protection services in RD and in the whole country. The overall test for parallelism revealed a statistically significant difference in trend rates between RD and the whole country (*p* = 0.009). From year 2013 onwards, the rate of the 0 to 17-year-old population referred to child protection services showed a significant decrease in RD (AAPC = −6.9; 95% CI: −10.8,−2.8; *p* = 0.013) compared to an increasing trend in the whole country (AAPC = 1.9; 95% CI: 0.6, 3.3; *p* = 0.020) (AAPC difference = 8.9; 95% CI: 6.2, 11.5; *p* < 0.001). In absolute numbers, during the year 2016, a total of 359 children in RD were referred to child protection services compared to 451 children in 2012, showing a decrease of 25%.

Before the year 2013, the rates had significantly increased in the whole country (AAPC = 4.1; 95% CI: 3.1, 5.2; *p* < 0.001), while no noticeable change was observed in RD (AAPC = −1.5; 95% CI: −4.6, 1.8; *p* = 0.373) (AAPC difference = 5.6; 95% CI: 2.2, 8.9; *p* = 0.001).

## Discussion

Let's Talk about Children Model was implemented in RD from 2012 to 2013. We analyzed referrals to child protection services based on the municipal register data and compared them with the national register data from 2009 to 2016. Referrals in RD decreased significantly between 2013 and 2016, while the national data showed a significant increase. We will first discuss the results and then the LT-SM.

The significant decrease in referrals in RD with the launch of LT-SM and the clear departure from the national trend suggest an association between the two. However, possible favorable contextual changes affecting child and family population might also have an impact on this outcome. RD has been an area with high unemployment. However, rather than a decrease, between 2012 and 2016 in RD, there was an increase in unemployment in the general population from 10 to 13%, and among individuals between 18 and 24 years from 14 to 20% (15). Unemployment is known to be a family stressor and related to use of child protection services (16). This was not reflected in the data, which may support the possible preventive impact of LT-SM. Furthermore, there were no other major concurrent projects targeted on families; however, as this was not a randomized controlled study, we cannot make definite causal conclusions. RD will continue to be evaluated to gain a more comprehensive picture in the future.

The decrease in referrals to child protection services suggests that families were getting the needed support before the family situation reached a breaking point. One Contact Service is likely to be crucial for convening LT Network meetings without delay. This might also explain why there was no early increase in the referrals as we expected.

We further hypothesized that the process would take years to yield results, but this was not the case. There are several possible reasons for this, including the expeditious implementation. The leaders of RD were deeply motivated to implement LT-SM and decisions could be made without delay. Training trainers also contributed to the rapid implementation and access to practical work with families.

### LT Service Model

LT-SM and its implementation present a novel approach regarding prevention, promotion, and service development. Rather than focusing on individual impact, it aims at the collective impact on population-level outcomes (4). Key aspects of the model are discussed as follows.

First, a commitment from both administrative and political leaders of the model is crucial. The implementation of LT-SM touches all corners of the service system and, through schools and kindergartens, the whole child population and their parents. The role of the Multiagent Management Group is important, which is a decision-making body including leaders from different service sectors. Problems are discussed and successes achieved around the same table.

The core of the Model is the agreement to promote children's well-being in everyday life in all developmental contexts. It is a goal that makes sense and can be scientifically argued and expressed in common language. It is the smallest common denominator for the fragmented services.

It is noteworthy to mention that the starting point for the process is the provision of practical support for children and families. Rather than waiting for the collaborative infrastructure to be developed before the corresponding work with families begins, LT-SM is built up through interaction with the experiences and needs identified in LT interventions with families. The work with families informs the change process and is its generator.

In conclusion, LT-SM recognizes that child and family well-being involves the whole ecological context. Our results are promising, but they are just a starting point from one district. Nevertheless, they encourage the implementation of the model in different types of municipalities and demand research on implementation strategies and impact mechanisms with long-term evaluation.

## Author Contributions

MN and TS developed the LT-Model and the implementation strategy. MN was responsible for taking the Model into practice and consulting and guiding the implementation process. TS was responsible for negotiating with the Ministries of Health and Social Affairs and Education concerning the needed approvals for the Model and writing up the manuscript with contributions from MN and SR. BA-A and JJ contributed to implementing the Model especially in the child protection and family social services. HK, was the motivational force for the implementation of the Model in Raahe District and instrumental for getting the political support. SR consulted implementation process especially relating to mental health services and made valuable contributions to the manuscript. HH had responsibility of statistical analyses and writing up the data analysis and results section of the manuscript. TR, on the impact of parental illness on children, provided important guidelines for implementing the Model in different services. All authors have approved the final version of the manuscript and agreed to be accountable for all aspects of the work in ensuring that questions related to the accuracy or integrity of any part of the work are appropriately investigated and resolved.

### Conflict of Interest Statement

The authors declare that the research was conducted in the absence of any commercial or financial relationships that could be construed as a potential conflict of interest. The handling editor declared a shared committee, though no other collaboration, with one of the authors TS at the time of the review.

## Footnotes

^1^https://www.copmi.net.au

## References

[1] Heino T Hyry S Ikäheimo S Kuronen M Rajala R Lasten Kodin ulkopuolelle sijoittamisen syyt, Taustat, Palvelut ja Kustannukset. HuosTa-hankkeen (2014–2015) Päätulokset. Terveyden ja hyvinvoinnin laitos (THL). Raportti3/2016. Helsinki. Available online at: https://thl.fi/documents/2616650/2646346/Lastensuojelu.pdf/e09efa65-c1a3-4e65-8039-6d716e63cdf0

[2] Ungar M Ghazinour M Richter J. Annual Research Review: what is resilience within the social ecology of human development? J Child Psychol Psychiatry (2013) 54:348–66. 10.1111/jcpp.12025 23215898

[3] Collishaw S Hammerton G Mahedy L Sellers R Owen MJ Craddock N. Mental health resilience in the adolescent offspring of parents with depression: a prospective longitudinal study. Lancet Psychiatry (2016) 3:49–57. 10.1016/S2215-0366(15)00358-2 26654748 PMC4703896

[4] Kania J Kramer M Collective impact. Stanford Soc Innov Rev. (2011) 9:36–41. Available online at: http://ssir.org/articles/entry/collective_impact

[5] Toikka S Solantaus T The effective family programme II: clinicians' experiences of training in promotive and preventative child mental health methods. Int J Ment Health Promot. (2006) 8:4–10. 10.1080/14623730.2006.9721746

[6] Solantaus T Toikka S Alasuutari M Beardslee WR Paavonen EJ Safety, feasibility and family experiences of preventive interventions for children and families with parental depression. Int J Ment Health Promot. (2009) 11:15–24. 10.1080/14623730.2009.9721796

[7] Punamäki R Paavonen J Toikka S Solantaus T. Effectiveness of preventive family intervention in improving cognitive attributions among children of depressed parents: a randomized study. J Fam Psychol. (2013) 27:683–90. 10.1037/a0033466 23978323

[8] Solantaus T Paavonen E Toikka S Punamäki R. Preventive interventions in families with parental depression: children's psychosocial symptoms and prosocial behaviour. Eur Child Adolesc Psychiatry (2010) 19:12. 10.1007/s00787-010-0135-3 20890622 PMC2988995

[9] Kallunki H Niemelä M Ala-Aho B Savela S Pesonen K Raahen seudulla lapsia ja perheitä tuetaan peruspalvelujen kautta. In: Rimpelä M Rimpelä M, editors. Säästöjä Lapsiperheiden Palveluremonteilla. Liiteraportti: Kuntien Kuvaukset Lapsiperheiden Palvelujen Kehittämisestä. Helsinki: Kunnallisalan kehittämissäätiö (2015). p. 89–96. Available online at: www.kaks.fi/sites/default/files/LIITERAPORTTI_1.pdf

[10] Solantaus T Niemelä M Perheterapia (2016) 32:21–33.

[11] Fixsen DL Blase KA Naoom SF Wallace F Core Implementation Components. Res Soc Work Pract. (2009) 19:531–40. 10.1177/1049731509335549

[12] Sotkanet.fi Statistical Information on Welfare and Health in Finland. Available online at: https://sotkanet.fi/sotkanet/en/index

[13] Jiang Z Qiu Z Hatcher J Joinpoint Trend Analysis of Cancer Incidence and Mortality Using Alberta data. Alberta Health Services. Available online at: http://www.cancerview.ca/idc/groups/public/documents/webcontent/csen_cproj_fy0910q3_joinpoint.pdf

[14] Kim H Fay MP Feuer EJ Midthune DN. Permutation tests for joinpoint regression with applications to cancer rates. Stat Med. (2000) 19:335–51. 10.1002/(SICI)1097-0258(20000215)19:3<335::AID-SIM336>3.0.CO;2-Z 10649300

[15] PoPSTer Pohjois-Pohjanmaan Sosiaali- ja Terveydenhuolto Osana Tulevaisuuden Maakuntaa (2017). Available online at: https://www.pohjois-pohjanmaa.fi/download/5051/popster-loppuraportti/pdf

[16] Brooks-Gunn J Schneider W Waldfogel J The great depression and the risk for child maltreatment. Child Abuse Neglect. (2013) 37:721–9. 10.1016/j.chiabu.2013.08.004 PMC3811916 24045057

